# 3,6-Dibromo-9-(4-bromo­benz­yl)-9*H*-carbazole

**DOI:** 10.1107/S1600536808042827

**Published:** 2009-01-08

**Authors:** Jian-lan Cui, Mei Duan, Liu-qing Cai

**Affiliations:** a School of Chemical Engineering and Environment, North University of China, Taiyuan 030051, People’s Republic of China; b Shanxi Provincial People’s Hospital, Taiyuan 030012, People’s Republic of China

## Abstract

The title compound, C_19_H_12_Br_3_N, was synthesized by *N*-alkyl­ation of 1-bromo-4-(bromo­meth­yl)benzene with 3,6-dibromo-9*H*-carbazole. There are two unique mol­ecules in the asymmetric unit. The carbazole ring system is essentially planar, with a mean deviation of 0.0402 Å for one mol­ecule and 0.0279 Å for the other. The carbazole planes are inclined to the benzene ring planes at dihedral angles of 58.3 (3) and 71.1 (3)° in the two mol­ecules.

## Related literature

For the pharmaceutical properties of carbazoles, see: Buu-Hoï & Royer (1950 ▶); Caulfield *et al.* (2002 ▶); Harfenist & Joyner (1983 ▶); Harper *et al.* (2002 ▶). For bond length data, see: Allen *et al.* (1987 ▶). For the synthesis of the title compound, see: Duan *et al.* (2005*a* ▶,*b* ▶); Smith *et al.* (1992 ▶). For related literature, see: Borzatta & Carrozza (1991 ▶). For a related structure, see: Cui *et al.* (2009 ▶).
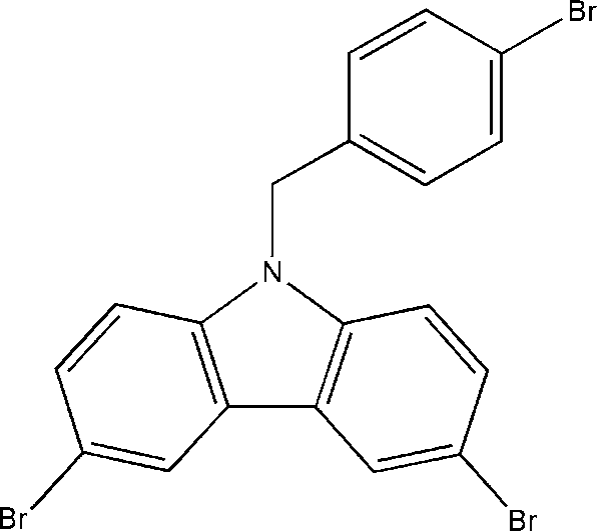


## Experimental

### 

#### Crystal data

C_19_H_12_Br_3_N
*M* *_r_* = 494.00
Monoclinic, 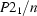

*a* = 9.4784 (19) Å
*b* = 17.132 (3) Å
*c* = 20.456 (4) Å
β = 98.16 (3)°
*V* = 3288.1 (11) Å^3^
*Z* = 8
Mo *K*α radiation
μ = 7.36 mm^−1^
*T* = 113 (2) K
0.08 × 0.02 × 0.02 mm

#### Data collection

Rigaku Saturn diffractometer
Absorption correction: multi-scan (*CrystalClear*; Rigaku/MSC, 2005 ▶) *T*_min_ = 0.591, *T*_max_ = 0.867
25134 measured reflections
7824 independent reflections
6058 reflections with *I* > 2σ(*I*)
*R*_int_ = 0.054

#### Refinement

*R*[*F* ^2^ > 2σ(*F* ^2^)] = 0.046
*wR*(*F* ^2^) = 0.103
*S* = 1.02
7824 reflections
415 parameters
H-atom parameters constrained
Δρ_max_ = 0.62 e Å^−3^
Δρ_min_ = −0.86 e Å^−3^

### 

Data collection: *CrystalClear* (Rigaku/MSC, 2005 ▶); cell refinement: *CrystalClear*; data reduction: *CrystalClear*; program(s) used to solve structure: *SHELXS97* (Sheldrick, 2008 ▶); program(s) used to refine structure: *SHELXL97* (Sheldrick, 2008 ▶); molecular graphics: *SHELXTL* (Sheldrick, 2008 ▶); software used to prepare material for publication: *SHELXTL*.

## Supplementary Material

Crystal structure: contains datablocks I, global. DOI: 10.1107/S1600536808042827/sj2546sup1.cif

Structure factors: contains datablocks I. DOI: 10.1107/S1600536808042827/sj2546Isup2.hkl

Additional supplementary materials:  crystallographic information; 3D view; checkCIF report

## Figures and Tables

**Fig. 1.**
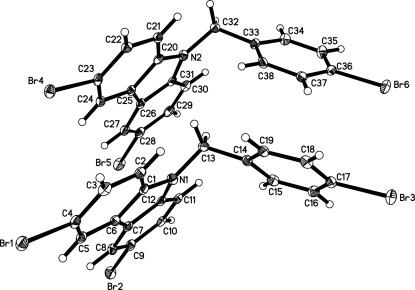
The asymmetric unit of (I) with displacement ellipsoids drawn at the 30% probability level.

## supplementary crystallographic information

### Comment

Carbazole derivatives substituted by *N*-alkylation possess valuable pharmaceutical properties (Buu-Hoï & Royer, 1950; Harfenist & Joyner, 1983; Caulfield *et al.*, 2002; Harper *et al.*, 2002). In this paper, we report the structure of 3,6-dibromo-9-(4-bromobenzyl)-9*H*-carbazole (I), which was synthesized by *N*-alkylation of 1-bromo-4-(bromomethyl)benzene with 3,6-dibromo-9*H*-carbazole. The carbazole ring system is essentially planar with mean deviations of 0.0402Å  for one molecule and 0.0279 Å for the other. The carbazole planes are inclined to the benzene ring planes at dihedral angles of 58.3 (3)° and 108.9 (3) ° respectively. The C—Br distances fall in the range 1.894 (6) to 1.911 (5) Å, consistent with the literature (Allen *et al.*, 1987).

### Experimental

The title compound was prepared according to the procedure of Duan *et al.* (2005*a*,*b*) from 3,6-dibromo-carbazole (Smith *et al.* 1992) and 1-bromo-4-(bromomethyl)benzene. Compound (I) (40 mg) was dissolved in mixture of chloroform (10 ml) and ethanol (5 ml) and the solution was kept at room temperature for 18 d. Natural evaporation of the solution gave colourless crystals suitable for X-Ray analysis. (m.p. 480–481 K).

### Refinement

All H atoms were included in the riding model approximation with C—H distances = 0.93 (aromatic) and 0.97 (methylene) Å, and with *U*_iso_(H) = 1.2x*U*_eq_(C).

### Crystal data

**Table:**

C_19_H_12_Br_3_N	*F*(000) = 1904
*M**_r_* = 494.00	*D*_x_ = 1.996 Mg m^−^^3^
Monoclinic, *P*2_1_/*n*	Melting point = 480–481 K
Hall symbol: -P 2yn	Mo *K*α radiation, λ = 0.71073 Å
*a* = 9.4784 (19) Å	Cell parameters from 6869 reflections
*b* = 17.132 (3) Å	θ = 1.6–28.0°
*c* = 20.456 (4) Å	µ = 7.36 mm^−^^1^
β = 98.16 (3)°	*T* = 113 K
*V* = 3288.1 (11) Å^3^	Prism, colorless
*Z* = 8	0.08 × 0.02 × 0.02 mm

### Data collection

**Table:**

Rigaku Saturn diffractometer	7824 independent reflections
Radiation source: rotating anode	6058 reflections with *I* > 2σ(*I*)
confocal	*R*_int_ = 0.054
ω scans	θ_max_ = 27.9°, θ_min_ = 1.6°
Absorption correction: multi-scan (*CrystalClear*; Rigaku/MSC, 2005)	*h* = −12→10
*T*_min_ = 0.591, *T*_max_ = 0.867	*k* = −20→22
25134 measured reflections	*l* = −26→26

### Refinement

**Table:**

Refinement on *F*^2^	Primary atom site location: structure-invariant direct methods
Least-squares matrix: full	Secondary atom site location: difference Fourier map
*R*[*F*^2^ > 2σ(*F*^2^)] = 0.046	Hydrogen site location: inferred from neighbouring sites
*wR*(*F*^2^) = 0.103	H-atom parameters constrained
*S* = 1.02	*w* = 1/[σ^2^(*F*_o_^2^) + (0.05*P*)^2^] where *P* = (*F*_o_^2^ + 2*F*_c_^2^)/3
7824 reflections	(Δ/σ)_max_ = 0.002
415 parameters	Δρ_max_ = 0.62 e Å^−^^3^
0 restraints	Δρ_min_ = −0.86 e Å^−^^3^

### Special details

**Table:**

Geometry. All e.s.d.'s (except the e.s.d. in the dihedral angle between two l.s. planes) are estimated using the full covariance matrix. The cell e.s.d.'s are taken into account individually in the estimation of e.s.d.'s in distances, angles and torsion angles; correlations between e.s.d.'s in cell parameters are only used when they are defined by crystal symmetry. An approximate (isotropic) treatment of cell e.s.d.'s is used for estimating e.s.d.'s involving l.s. planes.
Refinement. Refinement of *F*^2^ against ALL reflections. The weighted *R*-factor *wR* and goodness of fit *S* are based on *F*^2^, conventional *R*-factors *R* are based on *F*, with *F* set to zero for negative *F*^2^. The threshold expression of *F*^2^ > σ(*F*^2^) is used only for calculating *R*-factors(gt) *etc*. and is not relevant to the choice of reflections for refinement. *R*-factors based on *F*^2^ are statistically about twice as large as those based on *F*, and *R*- factors based on ALL data will be even larger.

### Fractional atomic coordinates and isotropic or equivalent isotropic displacement parameters (Å^2^)

**Table:**

	*x*	*y*	*z*	*U*_iso_*/*U*_eq_	
Br1	−0.14151 (4)	0.08974 (2)	0.20844 (2)	0.02780 (12)	
Br2	0.26336 (5)	0.39468 (3)	0.51841 (2)	0.03025 (12)	
Br3	0.10307 (5)	0.67522 (3)	0.01113 (2)	0.02914 (12)	
Br4	0.36237 (4)	0.08294 (2)	0.17850 (2)	0.02334 (11)	
Br5	0.71818 (6)	0.37880 (3)	0.50564 (2)	0.03361 (13)	
Br6	0.57775 (5)	0.68767 (2)	0.03165 (2)	0.02995 (12)	
N1	0.2671 (3)	0.36064 (19)	0.22607 (17)	0.0202 (7)	
N2	0.7685 (3)	0.35444 (19)	0.21649 (16)	0.0191 (7)	
C1	0.1733 (4)	0.2987 (2)	0.2121 (2)	0.0185 (8)	
C2	0.1328 (4)	0.2593 (2)	0.1533 (2)	0.0209 (9)	
H2	0.1683	0.2737	0.1150	0.025*	
C3	0.0377 (4)	0.1977 (2)	0.1532 (2)	0.0226 (9)	
H3	0.0097	0.1698	0.1145	0.027*	
C4	−0.0161 (4)	0.1774 (2)	0.2112 (2)	0.0228 (9)	
C5	0.0207 (4)	0.2165 (2)	0.2699 (2)	0.0218 (9)	
H5	−0.0178	0.2025	0.3076	0.026*	
C6	0.1174 (4)	0.2777 (2)	0.2708 (2)	0.0198 (9)	
C7	0.1859 (4)	0.3280 (2)	0.3219 (2)	0.0192 (8)	
C8	0.1804 (4)	0.3334 (2)	0.3900 (2)	0.0204 (9)	
H8	0.1203	0.3014	0.4104	0.024*	
C9	0.2673 (5)	0.3878 (2)	0.4256 (2)	0.0229 (9)	
C10	0.3593 (4)	0.4368 (2)	0.3975 (2)	0.0214 (9)	
H10	0.4161	0.4723	0.4237	0.026*	
C11	0.3660 (4)	0.4324 (2)	0.3301 (2)	0.0204 (9)	
H11	0.4266	0.4646	0.3103	0.025*	
C12	0.2786 (4)	0.3777 (2)	0.2932 (2)	0.0181 (8)	
C13	0.3542 (4)	0.3943 (2)	0.1802 (2)	0.0197 (9)	
H13A	0.3758	0.3538	0.1500	0.024*	
H13B	0.4437	0.4115	0.2050	0.024*	
C14	0.2862 (4)	0.4627 (2)	0.1401 (2)	0.0202 (9)	
C15	0.2893 (4)	0.5380 (2)	0.1667 (2)	0.0223 (9)	
H15	0.3293	0.5461	0.2103	0.027*	
C16	0.2335 (4)	0.6010 (2)	0.1286 (2)	0.0228 (9)	
H16	0.2349	0.6508	0.1466	0.027*	
C17	0.1758 (4)	0.5882 (2)	0.0636 (2)	0.0205 (9)	
C18	0.1704 (4)	0.5147 (2)	0.0362 (2)	0.0217 (9)	
H18	0.1308	0.5071	−0.0076	0.026*	
C19	0.2245 (4)	0.4523 (2)	0.0745 (2)	0.0204 (9)	
H19	0.2198	0.4024	0.0564	0.024*	
C20	0.6758 (4)	0.2931 (2)	0.1978 (2)	0.0175 (8)	
C21	0.6408 (4)	0.2566 (2)	0.1368 (2)	0.0187 (8)	
H21	0.6808	0.2732	0.1003	0.022*	
C22	0.5455 (4)	0.1955 (2)	0.1317 (2)	0.0195 (8)	
H22	0.5214	0.1699	0.0916	0.023*	
C23	0.4851 (4)	0.1720 (2)	0.1870 (2)	0.0202 (9)	
C24	0.5139 (4)	0.2079 (2)	0.2474 (2)	0.0195 (8)	
H24	0.4704	0.1918	0.2831	0.023*	
C25	0.6115 (4)	0.2698 (2)	0.25318 (19)	0.0169 (8)	
C26	0.6708 (4)	0.3185 (2)	0.3077 (2)	0.0202 (9)	
C27	0.6534 (5)	0.3220 (2)	0.3746 (2)	0.0224 (9)	
H27	0.5888	0.2895	0.3917	0.027*	
C28	0.7347 (4)	0.3749 (2)	0.4143 (2)	0.0225 (9)	
C29	0.8310 (5)	0.4252 (2)	0.3896 (2)	0.0253 (10)	
H29	0.8839	0.4601	0.4180	0.030*	
C30	0.8487 (4)	0.4238 (2)	0.3237 (2)	0.0240 (9)	
H30	0.9119	0.4575	0.3072	0.029*	
C31	0.7682 (4)	0.3698 (2)	0.2827 (2)	0.0192 (8)	
C32	0.8553 (4)	0.3959 (2)	0.1744 (2)	0.0214 (9)	
H32A	0.8769	0.3609	0.1399	0.026*	
H32B	0.9448	0.4106	0.2006	0.026*	
C33	0.7844 (4)	0.4684 (2)	0.14287 (19)	0.0191 (8)	
C34	0.8599 (5)	0.5385 (2)	0.1439 (2)	0.0239 (9)	
H34	0.9524	0.5411	0.1661	0.029*	
C35	0.7984 (5)	0.6039 (2)	0.1124 (2)	0.0256 (10)	
H35	0.8491	0.6505	0.1135	0.031*	
C36	0.6604 (4)	0.5997 (2)	0.0790 (2)	0.0218 (9)	
C37	0.5819 (4)	0.5316 (2)	0.0782 (2)	0.0219 (9)	
H37	0.4884	0.5298	0.0570	0.026*	
C38	0.6452 (4)	0.4661 (2)	0.1096 (2)	0.0217 (9)	
H38	0.5939	0.4198	0.1085	0.026*	

### Atomic displacement parameters (Å^2^)

**Table:**

	*U*^11^	*U*^22^	*U*^33^	*U*^12^	*U*^13^	*U*^23^
Br1	0.0208 (2)	0.0208 (2)	0.0402 (3)	−0.00171 (17)	−0.00107 (19)	0.00121 (19)
Br2	0.0424 (3)	0.0291 (3)	0.0198 (2)	0.0031 (2)	0.0065 (2)	0.00106 (18)
Br3	0.0335 (2)	0.0247 (2)	0.0277 (2)	0.00291 (18)	−0.0008 (2)	0.00693 (19)
Br4	0.0224 (2)	0.0197 (2)	0.0266 (2)	−0.00224 (16)	−0.00116 (18)	0.00099 (17)
Br5	0.0505 (3)	0.0309 (3)	0.0183 (2)	0.0093 (2)	0.0009 (2)	−0.00347 (19)
Br6	0.0369 (3)	0.0202 (2)	0.0314 (3)	0.00635 (18)	0.0000 (2)	0.00390 (19)
N1	0.0176 (16)	0.0219 (18)	0.0208 (18)	−0.0027 (14)	0.0016 (14)	0.0009 (15)
N2	0.0230 (17)	0.0165 (17)	0.0172 (17)	−0.0009 (14)	0.0005 (15)	−0.0007 (14)
C1	0.0189 (19)	0.0113 (19)	0.025 (2)	0.0029 (15)	0.0014 (17)	0.0007 (17)
C2	0.020 (2)	0.021 (2)	0.021 (2)	0.0048 (17)	−0.0010 (17)	0.0020 (17)
C3	0.019 (2)	0.021 (2)	0.025 (2)	0.0010 (17)	−0.0058 (18)	−0.0044 (18)
C4	0.021 (2)	0.017 (2)	0.029 (2)	0.0033 (17)	−0.0019 (18)	0.0031 (18)
C5	0.019 (2)	0.018 (2)	0.027 (2)	0.0041 (16)	0.0004 (18)	0.0041 (18)
C6	0.0169 (19)	0.021 (2)	0.022 (2)	0.0040 (16)	0.0018 (17)	0.0057 (17)
C7	0.0160 (19)	0.016 (2)	0.025 (2)	0.0037 (16)	0.0015 (17)	0.0015 (17)
C8	0.023 (2)	0.019 (2)	0.019 (2)	0.0013 (17)	0.0017 (17)	0.0035 (17)
C9	0.030 (2)	0.021 (2)	0.017 (2)	0.0076 (18)	0.0002 (18)	0.0029 (17)
C10	0.027 (2)	0.016 (2)	0.021 (2)	0.0080 (17)	0.0002 (18)	−0.0014 (17)
C11	0.022 (2)	0.016 (2)	0.022 (2)	0.0065 (16)	0.0002 (18)	0.0024 (17)
C12	0.0196 (19)	0.017 (2)	0.017 (2)	0.0086 (16)	−0.0009 (16)	0.0050 (16)
C13	0.0184 (19)	0.021 (2)	0.020 (2)	0.0002 (16)	0.0052 (17)	−0.0009 (17)
C14	0.018 (2)	0.021 (2)	0.021 (2)	−0.0001 (16)	0.0032 (17)	0.0038 (17)
C15	0.024 (2)	0.028 (2)	0.014 (2)	−0.0019 (18)	0.0016 (17)	−0.0034 (18)
C16	0.024 (2)	0.018 (2)	0.027 (2)	−0.0012 (17)	0.0052 (19)	−0.0030 (18)
C17	0.020 (2)	0.021 (2)	0.020 (2)	−0.0014 (17)	0.0027 (17)	0.0016 (17)
C18	0.020 (2)	0.029 (2)	0.015 (2)	−0.0055 (17)	−0.0019 (17)	−0.0013 (18)
C19	0.0183 (19)	0.020 (2)	0.023 (2)	−0.0026 (16)	0.0048 (17)	0.0007 (17)
C20	0.0151 (18)	0.016 (2)	0.020 (2)	0.0031 (15)	−0.0020 (16)	−0.0016 (16)
C21	0.021 (2)	0.018 (2)	0.017 (2)	0.0033 (16)	0.0025 (17)	0.0012 (16)
C22	0.022 (2)	0.019 (2)	0.017 (2)	0.0020 (16)	0.0012 (17)	−0.0016 (16)
C23	0.020 (2)	0.016 (2)	0.023 (2)	0.0023 (16)	−0.0022 (17)	0.0046 (17)
C24	0.025 (2)	0.016 (2)	0.018 (2)	0.0015 (17)	0.0036 (17)	0.0014 (16)
C25	0.0188 (19)	0.016 (2)	0.0150 (19)	0.0045 (15)	−0.0008 (16)	0.0033 (16)
C26	0.024 (2)	0.018 (2)	0.018 (2)	0.0071 (17)	0.0010 (17)	0.0048 (17)
C27	0.027 (2)	0.019 (2)	0.020 (2)	0.0053 (17)	0.0023 (18)	0.0021 (17)
C28	0.028 (2)	0.023 (2)	0.016 (2)	0.0100 (18)	−0.0016 (18)	0.0012 (17)
C29	0.028 (2)	0.017 (2)	0.028 (2)	0.0042 (18)	−0.005 (2)	−0.0080 (18)
C30	0.024 (2)	0.019 (2)	0.028 (2)	0.0024 (17)	0.0005 (19)	−0.0009 (18)
C31	0.0168 (19)	0.015 (2)	0.025 (2)	0.0070 (16)	−0.0004 (17)	0.0025 (17)
C32	0.020 (2)	0.020 (2)	0.026 (2)	0.0001 (16)	0.0059 (18)	0.0031 (18)
C33	0.021 (2)	0.020 (2)	0.015 (2)	0.0045 (16)	−0.0016 (16)	0.0012 (16)
C34	0.024 (2)	0.022 (2)	0.024 (2)	−0.0034 (17)	−0.0001 (18)	−0.0021 (18)
C35	0.032 (2)	0.018 (2)	0.026 (2)	−0.0057 (18)	0.001 (2)	−0.0025 (18)
C36	0.023 (2)	0.021 (2)	0.022 (2)	0.0050 (17)	0.0049 (18)	0.0012 (17)
C37	0.020 (2)	0.025 (2)	0.021 (2)	0.0013 (17)	0.0038 (17)	0.0027 (18)
C38	0.021 (2)	0.021 (2)	0.024 (2)	−0.0040 (17)	0.0058 (18)	0.0036 (18)

### Geometric parameters (Å, °)

**Table:**

Br1—C4	1.911 (4)	C16—C17	1.381 (6)
Br2—C9	1.908 (4)	C16—H16	0.9300
Br3—C17	1.907 (4)	C17—C18	1.377 (5)
Br4—C23	1.911 (4)	C18—C19	1.381 (6)
Br5—C28	1.899 (4)	C18—H18	0.9300
Br6—C36	1.900 (4)	C19—H19	0.9300
N1—C1	1.387 (5)	C20—C21	1.392 (5)
N1—C12	1.394 (5)	C20—C25	1.418 (5)
N1—C13	1.454 (5)	C21—C22	1.377 (5)
N2—C31	1.380 (5)	C21—H21	0.9300
N2—C20	1.389 (5)	C22—C23	1.397 (5)
N2—C32	1.457 (5)	C22—H22	0.9300
C1—C2	1.385 (6)	C23—C24	1.373 (6)
C1—C6	1.426 (5)	C24—C25	1.401 (5)
C2—C3	1.387 (5)	C24—H24	0.9300
C2—H2	0.9300	C25—C26	1.442 (6)
C3—C4	1.400 (6)	C26—C27	1.403 (6)
C3—H3	0.9300	C26—C31	1.422 (5)
C4—C5	1.375 (6)	C27—C28	1.377 (6)
C5—C6	1.392 (6)	C27—H27	0.9300
C5—H5	0.9300	C28—C29	1.400 (6)
C6—C7	1.436 (6)	C29—C30	1.382 (6)
C7—C8	1.404 (5)	C29—H29	0.9300
C7—C12	1.410 (5)	C30—C31	1.400 (6)
C8—C9	1.382 (6)	C30—H30	0.9300
C8—H8	0.9300	C32—C33	1.512 (5)
C9—C10	1.392 (6)	C32—H32A	0.9700
C10—C11	1.391 (6)	C32—H32B	0.9700
C10—H10	0.9300	C33—C38	1.396 (6)
C11—C12	1.398 (6)	C33—C34	1.396 (5)
C11—H11	0.9300	C34—C35	1.381 (6)
C13—C14	1.521 (5)	C34—H34	0.9300
C13—H13A	0.9700	C35—C36	1.389 (6)
C13—H13B	0.9700	C35—H35	0.9300
C14—C19	1.397 (6)	C36—C37	1.382 (5)
C14—C15	1.399 (6)	C37—C38	1.387 (6)
C15—C16	1.390 (6)	C37—H37	0.9300
C15—H15	0.9300	C38—H38	0.9300
			
C1—N1—C12	108.8 (3)	C18—C19—C14	121.1 (4)
C1—N1—C13	125.2 (3)	C18—C19—H19	119.4
C12—N1—C13	125.5 (3)	C14—C19—H19	119.4
C31—N2—C20	109.0 (3)	N2—C20—C21	130.3 (4)
C31—N2—C32	124.5 (3)	N2—C20—C25	108.7 (3)
C20—N2—C32	126.5 (3)	C21—C20—C25	121.0 (4)
C2—C1—N1	129.9 (4)	C22—C21—C20	118.7 (4)
C2—C1—C6	121.2 (4)	C22—C21—H21	120.7
N1—C1—C6	108.9 (4)	C20—C21—H21	120.7
C1—C2—C3	118.2 (4)	C21—C22—C23	119.8 (4)
C1—C2—H2	120.9	C21—C22—H22	120.1
C3—C2—H2	120.9	C23—C22—H22	120.1
C2—C3—C4	120.1 (4)	C24—C23—C22	123.2 (4)
C2—C3—H3	119.9	C24—C23—Br4	118.7 (3)
C4—C3—H3	119.9	C22—C23—Br4	118.0 (3)
C5—C4—C3	122.8 (4)	C23—C24—C25	117.3 (4)
C5—C4—Br1	119.2 (3)	C23—C24—H24	121.3
C3—C4—Br1	118.0 (3)	C25—C24—H24	121.3
C4—C5—C6	117.7 (4)	C24—C25—C20	120.0 (4)
C4—C5—H5	121.2	C24—C25—C26	133.1 (4)
C6—C5—H5	121.2	C20—C25—C26	106.9 (3)
C5—C6—C1	120.1 (4)	C27—C26—C31	119.9 (4)
C5—C6—C7	133.8 (4)	C27—C26—C25	133.8 (4)
C1—C6—C7	106.1 (3)	C31—C26—C25	106.3 (3)
C8—C7—C12	119.1 (4)	C28—C27—C26	117.9 (4)
C8—C7—C6	133.2 (4)	C28—C27—H27	121.0
C12—C7—C6	107.7 (3)	C26—C27—H27	121.0
C9—C8—C7	117.6 (4)	C27—C28—C29	122.2 (4)
C9—C8—H8	121.2	C27—C28—Br5	119.0 (3)
C7—C8—H8	121.2	C29—C28—Br5	118.8 (3)
C8—C9—C10	123.4 (4)	C30—C29—C28	121.0 (4)
C8—C9—Br2	118.2 (3)	C30—C29—H29	119.5
C10—C9—Br2	118.4 (3)	C28—C29—H29	119.5
C11—C10—C9	119.7 (4)	C29—C30—C31	117.8 (4)
C11—C10—H10	120.1	C29—C30—H30	121.1
C9—C10—H10	120.1	C31—C30—H30	121.1
C10—C11—C12	117.7 (4)	N2—C31—C30	129.6 (4)
C10—C11—H11	121.2	N2—C31—C26	109.1 (4)
C12—C11—H11	121.2	C30—C31—C26	121.2 (4)
N1—C12—C11	129.1 (4)	N2—C32—C33	113.5 (3)
N1—C12—C7	108.5 (4)	N2—C32—H32A	108.9
C11—C12—C7	122.4 (4)	C33—C32—H32A	108.9
N1—C13—C14	114.7 (3)	N2—C32—H32B	108.9
N1—C13—H13A	108.6	C33—C32—H32B	108.9
C14—C13—H13A	108.6	H32A—C32—H32B	107.7
N1—C13—H13B	108.6	C38—C33—C34	118.6 (4)
C14—C13—H13B	108.6	C38—C33—C32	121.2 (4)
H13A—C13—H13B	107.6	C34—C33—C32	120.2 (4)
C19—C14—C15	118.3 (4)	C35—C34—C33	120.6 (4)
C19—C14—C13	120.4 (4)	C35—C34—H34	119.7
C15—C14—C13	121.2 (4)	C33—C34—H34	119.7
C16—C15—C14	120.8 (4)	C34—C35—C36	119.5 (4)
C16—C15—H15	119.6	C34—C35—H35	120.2
C14—C15—H15	119.6	C36—C35—H35	120.2
C17—C16—C15	119.0 (4)	C37—C36—C35	121.2 (4)
C17—C16—H16	120.5	C37—C36—Br6	118.7 (3)
C15—C16—H16	120.5	C35—C36—Br6	120.1 (3)
C18—C17—C16	121.5 (4)	C36—C37—C38	118.7 (4)
C18—C17—Br3	119.8 (3)	C36—C37—H37	120.7
C16—C17—Br3	118.7 (3)	C38—C37—H37	120.7
C17—C18—C19	119.2 (4)	C37—C38—C33	121.3 (4)
C17—C18—H18	120.4	C37—C38—H38	119.3
C19—C18—H18	120.4	C33—C38—H38	119.3
			
C12—N1—C1—C2	−177.0 (4)	C31—N2—C20—C21	−179.7 (4)
C13—N1—C1—C2	−5.2 (7)	C32—N2—C20—C21	−0.8 (7)
C12—N1—C1—C6	2.9 (4)	C31—N2—C20—C25	1.6 (4)
C13—N1—C1—C6	174.7 (3)	C32—N2—C20—C25	−179.5 (3)
N1—C1—C2—C3	178.9 (4)	N2—C20—C21—C22	179.2 (4)
C6—C1—C2—C3	−1.0 (6)	C25—C20—C21—C22	−2.2 (6)
C1—C2—C3—C4	0.8 (6)	C20—C21—C22—C23	0.8 (6)
C2—C3—C4—C5	0.3 (6)	C21—C22—C23—C24	1.2 (6)
C2—C3—C4—Br1	−177.9 (3)	C21—C22—C23—Br4	−176.4 (3)
C3—C4—C5—C6	−1.1 (6)	C22—C23—C24—C25	−1.6 (6)
Br1—C4—C5—C6	177.1 (3)	Br4—C23—C24—C25	175.9 (3)
C4—C5—C6—C1	0.8 (6)	C23—C24—C25—C20	0.1 (6)
C4—C5—C6—C7	−175.9 (4)	C23—C24—C25—C26	−177.4 (4)
C2—C1—C6—C5	0.2 (6)	N2—C20—C25—C24	−179.4 (3)
N1—C1—C6—C5	−179.7 (3)	C21—C20—C25—C24	1.7 (6)
C2—C1—C6—C7	177.8 (4)	N2—C20—C25—C26	−1.2 (4)
N1—C1—C6—C7	−2.1 (4)	C21—C20—C25—C26	179.9 (3)
C5—C6—C7—C8	0.3 (8)	C24—C25—C26—C27	0.1 (8)
C1—C6—C7—C8	−176.8 (4)	C20—C25—C26—C27	−177.7 (4)
C5—C6—C7—C12	177.7 (4)	C24—C25—C26—C31	178.2 (4)
C1—C6—C7—C12	0.6 (4)	C20—C25—C26—C31	0.5 (4)
C12—C7—C8—C9	0.0 (6)	C31—C26—C27—C28	−1.3 (6)
C6—C7—C8—C9	177.1 (4)	C25—C26—C27—C28	176.7 (4)
C7—C8—C9—C10	−0.3 (6)	C26—C27—C28—C29	1.0 (6)
C7—C8—C9—Br2	−179.0 (3)	C26—C27—C28—Br5	−178.0 (3)
C8—C9—C10—C11	0.4 (6)	C27—C28—C29—C30	−0.1 (6)
Br2—C9—C10—C11	179.1 (3)	Br5—C28—C29—C30	179.0 (3)
C9—C10—C11—C12	−0.2 (6)	C28—C29—C30—C31	−0.7 (6)
C1—N1—C12—C11	176.2 (4)	C20—N2—C31—C30	176.9 (4)
C13—N1—C12—C11	4.4 (6)	C32—N2—C31—C30	−2.0 (6)
C1—N1—C12—C7	−2.5 (4)	C20—N2—C31—C26	−1.3 (4)
C13—N1—C12—C7	−174.3 (3)	C32—N2—C31—C26	179.8 (3)
C10—C11—C12—N1	−178.6 (4)	C29—C30—C31—N2	−177.6 (4)
C10—C11—C12—C7	−0.1 (6)	C29—C30—C31—C26	0.4 (6)
C8—C7—C12—N1	178.9 (3)	C27—C26—C31—N2	178.9 (4)
C6—C7—C12—N1	1.1 (4)	C25—C26—C31—N2	0.5 (4)
C8—C7—C12—C11	0.2 (6)	C27—C26—C31—C30	0.6 (6)
C6—C7—C12—C11	−177.6 (4)	C25—C26—C31—C30	−177.9 (4)
C1—N1—C13—C14	92.1 (5)	C31—N2—C32—C33	−88.0 (5)
C12—N1—C13—C14	−97.4 (5)	C20—N2—C32—C33	93.2 (5)
N1—C13—C14—C19	−101.7 (4)	N2—C32—C33—C38	−49.9 (5)
N1—C13—C14—C15	81.2 (5)	N2—C32—C33—C34	133.0 (4)
C19—C14—C15—C16	−0.5 (6)	C38—C33—C34—C35	−0.5 (6)
C13—C14—C15—C16	176.7 (3)	C32—C33—C34—C35	176.7 (4)
C14—C15—C16—C17	−0.7 (6)	C33—C34—C35—C36	−0.3 (6)
C15—C16—C17—C18	1.1 (6)	C34—C35—C36—C37	1.7 (6)
C15—C16—C17—Br3	−178.7 (3)	C34—C35—C36—Br6	−176.8 (3)
C16—C17—C18—C19	−0.4 (6)	C35—C36—C37—C38	−2.1 (6)
Br3—C17—C18—C19	179.5 (3)	Br6—C36—C37—C38	176.4 (3)
C17—C18—C19—C14	−0.9 (6)	C36—C37—C38—C33	1.3 (6)
C15—C14—C19—C18	1.3 (6)	C34—C33—C38—C37	−0.1 (6)
C13—C14—C19—C18	−175.9 (3)	C32—C33—C38—C37	−177.2 (4)
